# Scientist trust in medical research: a survey of authors from 28 countries

**DOI:** 10.3389/fmed.2025.1586885

**Published:** 2025-06-09

**Authors:** Arch G. Mainous, J. A. Kellie, Rachel E. Liu-Galvin, Barbara Durden, Valery M. Beau de Rochars

**Affiliations:** ^1^Department of Community Health and Family Medicine, University of Florida, Gainesville, FL, United States; ^2^Department of Health Services Research, Management, and Policy, University of Florida, Gainesville, FL, United States; ^3^Frontiers Publishing, Lausanne, Switzerland

**Keywords:** trust, research integrity, peer review, survey, international

## Abstract

**Importance:**

Peer-review is the lynchpin to research integrity, quality and trust in published health research findings.

**Objective:**

To evaluate the level of trust in peer-reviewed and non-peer-reviewed medical research among scientists who publish medical research.

**Methods:**

A survey was conducted of corresponding authors of papers accepted for publication in a peer-reviewed medical journal between September and December 2024 (*n* = 285). Survey questions focused on trust in the results in peer-review and non-peer-reviewed results. Deidentified data was provided to the current investigators for a secondary analysis. The level of press freedom in the country and whether the investigators in the country were oriented toward scientific papermills for publishing research was also evaluated.

**Results:**

Although 94% of the respondents have high trust in peer-reviewed research, a significant proportion (32.4%) have trust in non-peer-reviewed research. A majority (54.7%) believe that public trust in medical research findings is influenced by the reader’s political beliefs. The current peer review system is too slow (79%). Respondents from countries with a high prevalence of use of scientific papermills and low press freedom had more agreement that non-peer-reviewed research should be indexed than those from other countries (both *p* < 0.01). Authors who have published few papers are more trusting of non-peer-reviewed research (*p*.006) and more in agreement that non-peer-reviewed research should be indexed (*p*.015).

**Conclusion:**

Rebuilding the guardrails and trust in peer-review is necessary. A more streamlined peer-review system may be necessary to rebuild trust.

## Introduction

Trust in government health agencies in the US has been falling among the general public (1, 2). A lack of trust in government recommendations and health institutions among the public has been observed in multiple countries (3). This lack of trust among the public is associated with a perception of lack of consensus in science and changing perspectives (2).

Although the public has a diminishing lack of trust in the medical research and corresponding recommendations from health experts, the primary people who are producing the findings and interpreting the research are scientists. There is little data on the level of trust among scientists regarding the research published in scientific journals, and in particular, non-peer-reviewed research.

Research that has not been peer reviewed can influence practice and potentially cause harm (4, 5). Several authors have pointed out that a variety of preprints, non-peer reviewed articles that are widely disseminated, turned out to be fraudulent yet continued to be discussed long after they were retracted (6). In fact, some retracted information from preprints have been used to support political and proprietary interests. Further, papermills and predatory journals are flooding the scientific community with very poor quality research (7, 8).

Peer review is an effective strategy to decrease fraud and increase the quality of research (9). The gap in our knowledge about trust in medical research and the value of peer review by those individuals who are producing it is particularly important because it can point to a systemic problem with the original source of information that is disseminated to scientists, medical practitioners and the public.

In addition to the general gap in our knowledge of the perceptions of scientists, it is unclear whether any experiential or contextual variables may affect the perceptions of scientists toward trust in science in peer-reviewed journals and the rise and dissemination of non-peer-reviewed findings. Specifically, there is a knowledge gap as to whether factors such as a country’s press freedom or the embracing by peers of papermills as a way to author peer-reviewed publications affect researchers’ levels of trust in science and non-peer reviewed research (10–12).

Peer review of science is a bedrock strategy to ensure that results can be trusted. The evidence is limited regarding whether scientists have trust in non-peer-reviewed medical research. Realizing the gap in our knowledge of the level of trust in medical research among scientists who publish such research, we undertook a multinational survey of researchers who authored manuscripts in a peer-reviewed medical research journal.

## Methods

### Research design

*Frontiers in Medicine* conducted a survey in Qualtrics of corresponding authors of papers accepted for publication between September and December 2024. Non-responders received one follow-up prompt 1 week after the initial contact. 294 total responses were received with 285 indicating their country of residence. The sample represented 28 different countries. The countries are presented in Table 1. The response rate was 14%. A deidentified data set was shared with the current research team. This deidentified data was considered not human data by the Institutional Review Board at the University of Florida.

**TABLE 1:** Country of origin of respondents.

Country
Australia
Brazil
Canada
China
Colombia
Czech Republic
Ethiopia
Germany
Hungary
India
Italy
Japan
Kazakhstan
Mexico
Oman
Poland
Portugal
Serbia
Slovakia
South Korea
Spain
Sweden
Switzerland
Thailand
Turkey
United Arab Emirates
United Kingdom of Great Britain and Northern Ireland
United States of America

Survey questions focused on trust in the results in peer review journals, preprints (non-peer-reviewed), and the best sources for messaging scientific research, as well as whether results are perceived to be influenced by political concerns.

Two innovative variables were constructed to examine the cultural environment for research dissemination. The 2024 World Press Freedom Index (WPFI) score was computed for each country (10). This provides a general country level assessment of the amount of press freedom. Countries were categorized into two groups: high press freedom, operationalized as those with a WPFI score > 40, versus low press freedom, operationalized as those with a WPFI score < 40. The second variable was whether the country of residence was one which used scientific papermills for selling peer-reviewed studies. Countries were categorized into two groups: countries with a high orientation toward use of papermills, operationalized as >25% papermill contribution in lists of papermill created papers, versus countries with a low orientation toward use of papermills, categorized as <25% papermill contribution in prevalence of papermill created papers (11, 12).

Additionally, we examined how perceptions differed based on scientists’ research experience, considering both the length of time conducting research, and their experience publishing medical research papers in peer-reviewed journals. Length of time conducting research was categorized as 10 or more years conducting research versus less than 10 years conducting research. For number of medical research papers published in peer-reviewed journals, we classified authors as those who had 10 or more papers published versus those with fewer than 10 papers published.

Chi-square tests of independence were conducted to examine associations between both the cultural environment for research dissemination in authors’ country of residence and their research experience with their perceptions around current published medical research. When more than 20% of expected cell counts were <5, Fisher’s Exact Test was used. Analyses were conducted using SAS version 9.4 (SAS Institute Inc., Cary, NC). All *p*-values were two-sided, with *p* < 0.05 considered statistically significant.

## Results

Table 2 indicates the perception of respondents toward peer-reviewed and non-peer-reviewed medical research. Although 94% of the respondents have high trust in peer-reviewed research, a significant proportion (32.4%) have trust in non-peer-reviewed research. Yet, 74.2% of the total respondents feel that non-peer-reviewed research should not be disseminated to the public. Further, the current peer review system was not overwhelmingly endorsed, with 79% of the respondents agreeing that the peer-review system is too slow, and that this is driving the public to look toward more seemingly up to date sources of information.

**TABLE 2:** Trust in medical research among authors of peer-reviewed articles (*n* = 294).

Question/variable	Response	%
How long have you been conducting medical research?	<10 years	48.3
	>10 years	51.7
Approximately, how many medical research papers have you published in peer-reviewed journals?	<10 publications	56.1
	>10 publications	43.9
World Press Freedom Index (WPFI) score of scientist’s country of residence	WPFI score < 40	76.5
	WPFI score ≥ 40	23.5
Papermill contribution of scientists’ country of residence	>25% Papermill contribution (%)	74.4
	<25% Papermill contribution (%)	25.6
What is the primary characteristic that you use to decide if a journal is a predatory journal?	Appearance on a list of predatory journals	38.1
	Cost of article page charge	10.5
	Country of publisher	0.8
	Impact factor	50.6
Have you ever submitted a paper to what you consider to be a predatory journal?	Yes	21.9
	No	78.1
In your opinion, do you think that the public generally trusts medical research findings?	Yes	92.3
	No	7.7
Do you think that public trust in medical research findings is influenced by the reader’s political beliefs?	Yes	54.7
	No	45.3
Is the general public moving away from peer reviewed medical research to opinion leaders who share their political beliefs?	Yes	42.5
	No	57.5
How much trust do you have in medical research published in peer reviewed journals?	None/a little	6.0
	Some/a lot	94.0
How much trust do you have in medical research that has not been peer reviewed?	None/a little	67.6
	Some/a lot	32.4
Are you familiar with the concept of preprints (i.e., release of studies before peer review) through outlets like MedRxiv or BioRxiv?	Yes	54.1
	No	45.9
(*Among those who knew what preprints are*) Are preprints undermining trust in medical research findings?	Yes	29.0
	No	71.0
Should PubMed or other databases index non-peer-reviewed studies?	Yes	45.3
	No	54.7
Please indicate the extent to which you agree with the following statements:		
Non-peer-reviewed medical research should not be allowed to be disseminated to the public.	Completely agree/somewhat agree	74.2
	Somewhat disagree/completely disagree	25.8
Current opinion leaders on social media are good at getting technical information on medical findings into messages that patients can understand.	Completely agree/somewhat agree	79.0
	Somewhat disagree/completely disagree	21.0
Physicians should be the primary strategy for communicating health recommendations from government agencies to patients.	Completely agree/somewhat agree	90.6
	Somewhat disagree/completely disagree	9.4
When findings from medical research change often, this makes it more difficult for patients to trust the recommendations.	Completely agree/somewhat agree	87.6
	Somewhat disagree/completely disagree	12.4
The peer reviewed system for medical research is too slow and so patients look toward other sources of up to date information.	Completely agree/somewhat agree	79.0
	Somewhat disagree/completely disagree	21.0

Respondents from countries with a high prevalence of use of scientific papermills and low press freedom had more agreement that non-peer-reviewed research should be indexed than those from other countries (both *p* < 0.01) (Table 3a). Moreover, respondents from countries with low press freedom and those from countries with high prevalence of use of papermills were significantly more likely to agree that opinion leaders are good avenues for disseminating research compared to their counterparts (both *p* < 0.0001).

**TABLE 3a:** Comparison of trust in medical research among authors of peer-reviewed articles by cultural environment for research dissemination in their country of residence (*n* = 285).

Question	Response	WPFI score < 40 (%)	WPFI score ≥ 40 (%)	*P*-value	>25% papermill contribution (%)	<25% papermill contribution (%)	*P*-value
Is the general public moving away from peer reviewed medical research to opinion leaders who share their political beliefs?	Yes (vs. No)	41.9	46.6	0.528	41.4	47.5	0.405
How much trust do you have in medical research that has not been peer reviewed?	Some or a lot (vs. None or a little)	34.8	22.4	0.078	35.4	21.3	0.042*
Should PubMed or other databases index non-peer-reviewed studies?	Yes (vs. No)	50.0	29.3	0.006*	50.3	29.5	0.005*
Please indicate the extent to which you agree with the following statements:							
Non-peer-reviewed medical research should not be allowed to be disseminated to the public.	Agree (vs. Disagree)	75.4	70.7	0.475	75.6	70.5	0.435
Current opinion leaders on social media are good at getting technical information on medical findings into messages that patients can understand	Agree (vs. Disagree)	88.3	50.0	<0.0001*	88.7	50.8	<0.0001*
Physicians should be the primary strategy for communicating health recommendations from government agencies to patients	Agree (vs. Disagree)	91.2	87.9	0.462	91.1	88.5	0.563

*Indicates statistical significance.

Table 3b indicates that authors who have published few papers are more trusting of non-peer-reviewed research (p.006) and more in agreement that non-peer-reviewed research should be indexed (p.015). Years of experience was not significantly related to any of the perceptions.

**TABLE 3b:** Comparison of trust in medical research among authors of peer-reviewed articles by research experience (*n* = 285).

Question	Response	<10 years (%)	>10 years (%)	*P*-value	<10 publications (%)	>10 publications (%)	*P*-value
Is the general public moving away from peer reviewed medical research to opinion leaders who share their political beliefs?	Yes (vs. no)	46.3	38.7	0.225	42.8	42.2	0.925
How much trust do you have in medical research that has not been peer reviewed?	Some or a lot (vs. none or a little)	35.0	29.8	0.390	39.3	22.6	0.006*
Should PubMed or other databases index non-peer-reviewed studies?	Yes (vs. no)	46.7	43.8	0.648	51.8	36.0	0.015*
Please indicate the extent to which you agree with the following statements:							
Non-peer-reviewed medical research should not be allowed to be disseminated to the public.	Agree (vs. disagree)	75.0	73.5	0.794	77.4	70.0	0.198
Current opinion leaders on social media are good at getting technical information on medical findings into messages that patients can understand	Agree (vs. disagree)	77.6	80.3	0.606	84.2	72.0	0.024*
Physicians should be the primary strategy for communicating health recommendations from government agencies to patients	Agree (vs. disagree)	92.2	88.9	0.382	94.0	86.0	0.039*

*Indicates statistical significance.

## Discussion

In this multinational survey of published authors of medical research, a majority agreed that politics plays a role in the public’s interpretation and acceptance of medical research. The findings reinforce the need to strengthen the peer-reviewed system to ensure research integrity and appropriate reporting of findings. Individuals who have been less successful in publishing articles are more positive about breaking down the peer-review guardrails for research integrity and both indexing non-peer-reviewed research and trusting in its veracity. Additionally, the contextual environment of the country of residence, like press freedom, plays a significant role in medical researchers’ trust in non-peer-reviewed research and trust in who disseminates the findings.

Rebuilding the guardrails and trust in peer-review is necessary. The majority of preprints, non-peer-reviewed abstracts and papers, do not end up going through the peer-review system within a year of posting (13, 14). Further, many of those abstracts provide inaccurate and incomplete interpretations of the data to fit a belief system endorsed by the authors. Thus, medical decision making would be negatively affected if we were to rely solely on non-peer-reviewed findings. Unfortunately, 79% of the respondents felt that the peer-review system is too slow and that this is driving the public to look toward more seemingly up to date sources of information. There has been a huge rise in the number of journals and the corresponding requests to review papers, which is a voluntary task (15). This has made it hard to find reviewers for many papers, thereby slowing down the peer review process. Just pushing scientists and the public toward the current peer-reviewed system may not yield positive results. A more streamlined peer-review system may be necessary.

There are some limitations to this study. The respondents were from 28 different countries, but the pool was drawn from only one journal. Although, we did assess how many peer reviewed articles the individuals have authored it is unclear in which journals the respondents have published. A second limitation of our study is although response rates have been dropping for many important surveys like the Health Information National Trends Survey (HINTS) (the response rate for the 2022 HINTS was 28%) our response rate was 14% (16). It is possible that there may be some bias in the responses between the respondents and non-respondents. Third, notwithstanding that we used two innovative variables defining the countries, local cultural mores were not collected.

In conclusion, authors of medical research have a high degree of trust in peer-reviewed medical research, but they acknowledge a variety of threats to having medical decision making and public awareness and acceptance of health recommendations based on peer-reviewed research. Political influence and the slowness of the system are making non-peer-reviewed research more attractive to some researchers and the public even with the potential risks of relying upon unverified findings. A strengthened peer-review system is needed to protect research integrity and population health.

## Data Availability

The raw data supporting the conclusions of this article will be made available by the authors, without undue reservation.
